# Antimicrobial resistance: One Health approach

**DOI:** 10.14202/vetworld.2022.743-749

**Published:** 2022-03-28

**Authors:** Maria Elena Velazquez-Meza, Miguel Galarde-López, Berta Carrillo-Quiróz, Celia Mercedes Alpuche-Aranda

**Affiliations:** Centro de Investigación sobre Enfermedades Infecciosas, Instituto Nacional de Salud Pública, Cuernavaca, Morelos, Mexico

**Keywords:** antimicrobials, human, animals, plants

## Abstract

In this research, a review of antimicrobial resistance (AMR) is conducted as part of the One Health approach. A review of publications, which included “antimicrobial resistance” and “One Health,” was conducted. Among the global health problems, AMR is the one that most clearly illustrates the One Health approach. AMR is a critical global problem affecting humans, the environment, and animals. This is related to each of these three components due to the irresponsible and excessive use of antimicrobials in various sectors (agriculture, livestock, and human medicine). Improper management of antimicrobials, inadequate control of infections, agricultural debris, pollutants in the environment, and migration of people and animals infected with resistant bacteria facilitate the spread of resistance. The study aimed to analyze the problem of AMR from a health perspective to analyze the different actors involved in One Health.

## Introduction

Among global health problems, antimicrobial resistance (AMR) is the one that best illustrates the One Health approach. The One Health approach is defined as a joint effort of various disciplines that come together to provide solutions for human, animal, and environmental health [1]. AMR is linked to each of these three components due to the irresponsible and excessive use of antimicrobials in various sectors (agriculture, cattle raising, and human medicine) [2,3]. Under the pressure of antimicrobial selection, bacteria acquire resistance genes and mobile genetic elements that can spread to other bacteria of the same or different genus. When bacteria acquire resistance to antimicrobials, they also acquire a greater ability to proliferate in animals, humans, and the natural world [4]. Mismanagement of antimicrobials, inadequate infection control, agricultural debris, contaminants in the environment, and migration of people and animals infected with resistant bacteria facilitate the spread of resistance [4,5].

**Figure-1:**
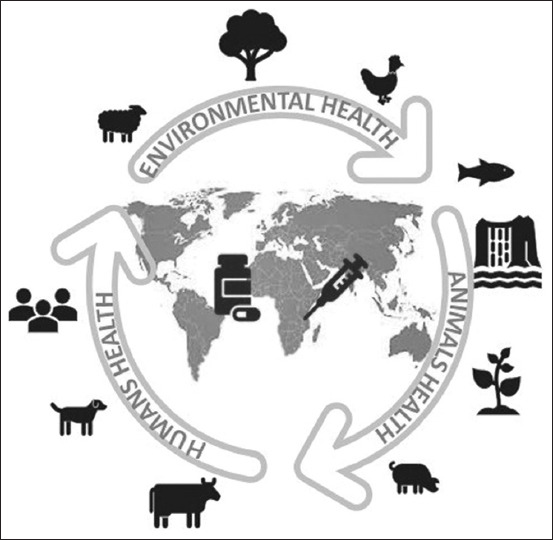
Schematic representation of the antimicrobial resistance under the perspective One Health [Source: Figure prepared by the authors].

AMR is a critical global problem that affects human, environmental, and animal health (Figure-1). Because AMR is a complex problem, it is necessary to look at it from different disciplines to frame it within the One Health approach [6-8].

The “One Health” approach originated in the 19^th^ century, when Rudolf Virchow introduced the term “zoonosis,” which encompasses the relationship between human and animal health [9]. Subsequently, Calvin Schwabe made important contributions to the disciplines of public health, epidemiology, and tropical medicine. He introduced the concept of one medicine, reaffirming the close relationship between human medicine and animal medicine [10]. In 2004, the Wildlife Conservation Society held a congress to discuss the implications of disease transmission in wildlife, domestic animals, and humans. Based on this meeting, the Manhattan Principles were established to combat infectious diseases and maintain the balance of ecosystems [11].

The study aimed to analyze the problem of AMR from a health perspective to analyze the different actors involved in One Health.

### Antimicrobial Resistance from the One Health Approach

Of particular concern is the rapid global spread of multidrug-resistant bacteria causing infections that cannot be treated with current antimicrobials. In 2019, the World Health Organization (WHO) identified 32 antimicrobials in hospital development, of which only six were classified as innovative. The lack of antimicrobials is affecting global health systems. Currently, infections caused by antimicrobial-resistant microorganisms are difficult to treat because antimicrobials are increasingly ineffective against these infections resulting in higher mortality rates. New antimicrobials are needed to control infections caused by the major pathogens outlined by the WHO. Now, unless the way current antimicrobials are used has not changed, these new antimicrobials will suffer the same fate as the current antimicrobials and become ineffective. Antimicrobial retention AMR has a significant impact on national economies and their health systems, as it affects the productivity of patients or caregivers due to prolonged hospital stays with high economic costs. The main factor of AMR includes improper and excessive use of antimicrobials; lack of access to clean water, sanitation, and hygiene for humans and animals; poor infection prevention and control measures in hospitals; poor access to medicines and vaccines; lack of awareness and knowledge; and irregularities with legislation [12].

AMR represents a global public health problem, for which epidemiological surveillance systems have been established, aiming to promote collaborations directed at the well-being of human and animal health and the balance of the ecosystem. Several international organizations (The World Organisation for Animal Health [OIE], WHO, and the Food and Agriculture Organization of the United Nations [FAO]) have joined forces to develop a Global Action Plan on Antimicrobial Resistance-WHO [12]. Action taken in this plan included understanding the AMR from surveillance and research. The advisory group established guidelines for AMR surveillance to ensure all countries implement integrated surveillance, which will cover the use and consumption of antimicrobials in the human and animal population. These guidelines will provide a clear understanding of how AMR spreads in different settings and specific areas. It will allow to study the correlation between AMR and antimicrobial use in a different setting (animals, humans, and environment) and to assess the effect of interventions within and between sectors [13,14,15].

The Interagency Coordination Group on Antimicrobial Resistance (WHO, OIE, and FAO) on AMR presented to the UN Secretary-General in April 2019 its report entitled “We can’t: securing the future against drug-resistant infections.” In addition, a joint tripartite secretariat (FAO, OIE and WHO) was established, based at WHO, to promote multi-stakeholder collaboration on AMR. New governance structures were agreed upon, such as the global AMR leaders group, the independent AMR action reporting group and the multi-lateral collaborative platform. One of the strategies to raise awareness of the AMR problem was the launch of the “Global Antimicrobial Awareness Week”. From 2020, it is called “World Antimicrobial Awareness Week,” referring to all antimicrobials: antibiotics, antifungals, antiparasitics, and antivirals. It is a global campaign that aims to raise awareness of AMR worldwide and encourage best practices among the general population, healthcare workers, and policymakers to curb the evolution and spread of drug-resistant infections.

In 2015, WHO launched the Global Antimicrobial Resistance Surveillance System (GLASS) to fill knowledge gaps and guide strategies at all levels. GLASS was created to progressively integrate surveillance data on antimicrobials used in humans, track antimicrobial use, and understand the role of AMR in the food chain and the environment. It provides a standardized approach to collecting, analyzing, interpreting, and sharing data by country, region, and area, allowing you to monitor the status of new or existing national surveillance systems, emphasizing the representativeness and quality of the data collected. In 2017, WHO developed the list of priority pathogens to guide research and development of new antimicrobials, diagnostic tools, and vaccines. WHO annually reviews preclinical and clinical antimicrobial development projects to assess their progress against the priority pathogens. This list will be updated in 2022. Additionally, the Global Alliance for Antibiotic Research and Development is a joint initiative of WHO and the Drugs for Neglected Diseases Initiative, supporting research and development through public–private partnerships. The partnership aims to develop and implement five new treatments against drug-resistant bacteria identified by WHO as the greatest threat by 2025 [12].

### Antimicrobial use in Humans, Animals, and Plants

Some antimicrobials were used for decades before resistance developed, whereas other antimicrobials developed resistance in a much shorter time. Antimicrobials with slow resistance development, especially vancomycin, were highly valued for their continued ability to treat infections that could not be treated with other commonly used antimicrobials. Today, the increasing vancomycin resistance is a concern as certain strains of bacteria that previously posed a relatively minor health risk, such as vancomycin-resistant enterococci, contribute greatly to mortality and morbidity, particularly in hospitals [6].

Antimicrobials have various uses in animals, including pets, farmed fish in aquaculture systems, bees, and farm animals. Antimicrobials are used for various purposes (therapeutic, prophylactic, and development promoters) and play an important role in animal production. The volume of antimicrobials used in animals worldwide is estimated to be greater than in humans. Most classes of antimicrobials used in humans are prescribed for animals, including classes of antimicrobials vital to human medicine, such as broad-spectrum beta-lactams and quinolones [16].

Some antimicrobials used in humans and animals (tetracycline, triazoles, and streptomycin) are used therapeutically in plants. AMR can be easily transferred between and within different ecosystems and populations; resistant zoonotic bacteria can be found in the soil; and from there, they can infect plants, vegetables, and fruits. It has been documented that the use of antimicrobials in agriculture induces antibiotic-resistant fungi transmitted from the environment to humans [17]. There are some classes of antimicrobials for human use only (carbapenems) and others for animal use only (flavophospholipol and ionophores) [18,19]. Other antimicrobials for clinical use, such as tetracycline and streptomycin, are used for prophylaxis and treatment against bacteria that cause fruit infection [20]. Antimicrobials doses used in aquaculture may be higher than those prescribed for livestock. Residues of antimicrobials remain in fish products and can remain in aquatic environments for a long time through excreta. These residues spread rapidly in water bodies, exerting selective pressure [21]. Antimicrobials are widely used as growth promoters, which is the main reason for the large volumes of antimicrobials used in the animal food industry [22].

## Antibiotic use from the One Health Approach

### Examples of antibiotics used in humans and animals

#### Colistin

Colistin (polymyxin B) is a highly bactericidal antibiotic that has been used by humans and animals for decades, but its systemic administration causes nephrotoxicity [23]. The use of this antibiotic is limited to treating patients with skin infections or cystic fibrosis. However, the frequency of systemic administration of colistin has been increased for treating infections caused by carbapenem-resistant bacteria (*Escherichia coli* and *Pseudomonas aeruginosa*) [24-26]. In countries where colistin is used to treat infections or as a growth promoter in animal production, excessive use of this antibiotic relative to human doses has been observed, although this varies by country [24,27,28].

Colistin resistance was initially chromosomally encoded, but in 2015, the plasmid-mediated *mcr-1* gene was reported to cause colistin resistance in *E*. *coli* strains, isolated from samples of foods, animals, and blood cultures in China [29]. Additionally, the *mcr-1* gene is present in other bacterial genera (*P*. *aeruginosa*, *Enterobacter* spp., and *Klebsiella pneumoniae*) [29]. Other studies have reported the presence of the *mcr-1* gene in various parts of the world in bacteria isolated from environmental, animal, and surface water samples [29,30].

The emergence of colistin resistance indicates that resistance can be increased further with the use of high doses of antimicrobials as growth promoters or to treat infections. The same problem was observed when avoparcin was used as a growth promoter. Also, vancomycin, another glycopeptide used in methicillin-resistant *Staphylococcus aureus* (MRSA), has resulted in resistance to severe infections caused by enterococci [31,32].

### Third-generation cephalosporins

These beta-lactam antibiotics are widely used in animals and humans; ceftriaxone, cefotaxime, and other cephalosporins are used to treat various infections in humans: urinary tract, abdominal, pulmonary and bloodstream infections [33]. Because of its usefulness in treating bacteria associated with AMR, this group of antibiotics has been classified as “ significant” to health [34].

Ceftiofur is the most widely used veterinary cephalosporin, followed by cefoperazone, cefovecin, and cefpodoxime. Ceftiofur is approved in many countries to treat bacterial infections, primarily in animals for human consumption. Its application is limited to parenteral use and is used in animals individually or in groups. Depending on the animal species, ceftiofur is used to treat meningitis, septicemia, pneumonia, septic arthritis, polyserositis, and metritis, among other types of infection. It is also sometimes used for respiratory diseases, prophylaxis in beef cattle, or preventing *E*. *coli* infections in broiler chickens [35].

In Europe, which has recorded data on antibiotic use for many years, approximately 18 tons of third- and fourth generation cephalosporins were used in 2017, mainly in animals intended for human consumption [28]. This represented approximately 0.2% of the total antimicrobial use in animals in Europe. In the USA, the total cephalosporin use for animals was approximately 31.44 tons in 2018 [36].

Extended-spectrum beta-lactamases are capable of inactivating third-generation cephalosporins (ceftriaxone, cefotaxime, ceftazidime) and aztreonam. The genes that code for this resistance is transferred by plasmids and transposons. AmpC beta-lactamases were first detected in chromosomes and later expressed in plasmids, indicating the presence of horizontal gene transfer among enterobacteria [37]. Resistance to *E*. *coli* and *K*. *pneumoniae* cephalosporin strains that cause serious infections are now reported in many countries. This has led to increased use of several existing antimicrobials, such as carbapenems [38].

Ceftiofur is primarily used to prevent *E. coli* infections and yolk sac infections [39,40]. This treatment has been shown to select strains of *Salmonella* resistant to cephalosporins; this bacterium causes serious diseases in humans and is associated with the consumption of contaminated poultry products [39-41]. Monitoring by the Canadian Integrated Program for AMR Surveillance revealed a temporal correlation between ceftiofur and ceftriaxone resistance in *Salmonella* Heidelberg strains isolated from poultry and humans [42].

### Fluoroquinolones

Fluoroquinolones are a family of broad-spectrum agents used to treat respiratory and urinary tract infections and are active against many Gram-positive and Gram-negative bacteria. Resistance to fluoroquinolones is caused by loss of porins, by the presence of efflux pumps or by alteration of the target sites of DNA gyrase and topoisomerase IV. Resistance to horizontally applied quinolones was first described in 1998. The *qnr* gene, located in a mobile genetic element, is responsible for this type of resistance [43].

Fluoroquinolones are another class of important antimicrobials where resistance has emerged among *Campylobacter jejuni* isolate medications in poultry [44]. In Australia, where fluoroquinolones have never been approved for use in animal feed, strain resistance to fluoroquinolones is rare [45]. The use of fluoroquinolones in livestock has been identified as a critical area because of the importance of these antimicrobials in treating human infections. In 2017, approximately 216 tons of fluoroquinolones were used in Europe, mainly in animals meant for human consumption [28]. This represented approximately 2.4% of all antimicrobials used in animals in Europe, and the total fluoroquinolone used in animals in the USA was approximately 23.3 tons in 2018 [36].

Given the importance of cephalosporins and fluoroquinolones in treating humans and selecting drug-resistant bacteria that can be transmitted from animals to humans, the use of cephalosporins and fluoroquinolones must be restricted [39-45].

### Implications for public and animal health

AMR reduces the effectiveness of antimicrobial therapy and often increases the cost incidence and severity of infection [3,46]. Currently, there is scientific evidence that the indiscriminate use of antimicrobials in the veterinary field has led to the emergence of resistant bacteria that cause infections in humans, particularly in *Enterococcus* spp., *Campylobacter* spp., *Salmonella* spp., and *E*. *coli* strains [31,42,44].

From the perspective of the One Health approach, resistance to cephalosporins is a good example of how antibiotics play an important role in both animal and human health. The main concern with the use of antimicrobials used in animals for therapeutic or prophylactic purposes is the spread of AMR [40]. Fluoroquinolones used in food animals are associated with quinolone resistance in Salmonella strains. Resistance to carbapenemics has been observed in Salmonella strains due to routine administration of ceftiofur [47,48].

*E*. *coli* is an important pathogen that is a common cause of bacterial infections, such as enteritis and urinary tract and bloodstream infections. AMR is a rapidly growing problem associated with *E*. *coli* infections in animals and humans, and this problem is best documented in human infection isolates, particularly in developing countries [49,50].

Severe staphylococcal infections in communities and hospital environments are mainly caused by MRSA strains responsible for various infections (skin and wound infections and bacteremia, among others) [51]. *S*. *aureus* and other staphylococcal species also affect animals [52,53]. This bacterium causes mastitis in cattle and skin infections in pigs and pets. Pathogenic MRSA strains in humans have emerged in different animal species. It is transmitted to humans through close contact with animals that are carriers of these strains [54,55].

### One Health approach to combat AMR

The One Health approach is fully integrated into global efforts to address the problem of AMR. Among the many obstacles to overcome are the competing interests of multiple economic sectors and organizations involved in animal, human, and environmental health. These actors need to agree on key priorities for action, the best ways to monitor AMR and control infections, and policies that should govern antimicrobial use. Key strategies for addressing AMR from the One Health approach are described:

1. Conduct a global public awareness campaign to educate our society about the harm caused by the overuse and misuse of antimicrobials. Implementing effective public campaigns can reduce the number of antimicrobials prescribed.
2. Improve and strengthen hygiene measures and prevent the spread of infections. By improving healthcare systems and living standards, we can significantly reduce the demand for antimicrobial and thus reduce the risk of the emergence of the new resistant strain.
3. Reduce the unnecessary use of antimicrobials in agriculture and their dissemination to the environment. Globally, the largest amounts of antimicrobials are consumed in agriculture and aquaculture. The use of antimicrobials as prophylaxis and growth promoters should be considered dangerous and unnecessary. Furthermore, it has been documented that animal excretes a significant percentage (75%–90%) of antimicrobials without being metabolized and dispersed into the environment.
4. Improve global surveillance of drug resistance. The medical and scientific community needs a clear understanding of current and historical data on AMR to clarify the new mechanisms of resistance acquisition, to know definitively current cases, and to predict future threats. To do so requires a better understanding of three areas: antibiotic consumption in humans and animals, current rates of antibiotic resistance, and a better understanding of the molecular basis of AMR.
5. Promote new and rapid clinical diagnoses. Misdiagnoses made in public or private hospitals lead to unnecessary antibiotic prescriptions. The development of rapid and accurate diagnostic tests will allow clinicians to administer antimicrobials to patients who need them.
6. Promote the development and use of vaccines and alternatives. The development of vaccines directed against antibiotic-resistant bacteria that cause serious infections will reduce the number of infected patients needing antimicrobial treatment. Additional investments are currently needed to develop new vaccines and alternatives to antimicrobials such as phage therapy, probiotics, antibodies, and lysins, among others.
7. Recognize and increase the number of people working with infectious diseases. Addressing AMR requires skilled professionals such as microbiologists, pharmacists, infectious disease specialists, nurses, infection control specialists, veterinarians, and epidemiologists. To do this, countries must invest in the training of this human resource.
8. A global innovation fund for early-stage research on new treatments. More public and private investments in drug discovery research are needed to develop new treatments. A global innovation fund is needed to support research that is not commercially attractive.
9. Create better incentives to promote investment in new drugs and in the improvement of existing drugs. The development of new antimicrobials is unattractive to pharmaceutical companies because there are still relatively effective antimicrobials on the market. It is difficult to predict exactly how and when AMR will develop, creating uncertainty for pharmaceutical companies when making business decisions.
10. Build a global coalition for real action against AMR. Global action is essential to make significant progress in the fight against AMR. Putting AMR on the international political agenda and addressing it using One Health approach is important to effect change [3].

## Conclusion

To combat AMR, it is important to support a “One Health” approach (human, animal, plant, and environmental health). This requires accelerating global progress, innovating to secure the future, collaborating for more effective action, investing in sustainable response, and strengthening global governance and accountability. Most classes of antibiotics are available for use in humans and animals. AMR can be reduced when antimicrobials are used only as a treatment, rarely for prophylaxis, and never as growth promoters. Success will require strict and efficient control of the types and amounts of antimicrobials used in medical practice and monitoring and controlling the proliferation of resistant bacteria that spread to the environment.

## Authors’ Contributions

CMAA: Suggested the concept of the study. MEVM: Drafted and critically revised the manuscript. MGL and BQC: Reviewed and revised the manuscript. All authors read and approved the final manuscript.
